# Identification of novel biomarkers of hepatocellular carcinoma by high‐definition mass spectrometry: Ultrahigh‐performance liquid chromatography quadrupole time‐of‐flight mass spectrometry and desorption electrospray ionization mass spectrometry imaging

**DOI:** 10.1002/rcm.8551

**Published:** 2019-11-06

**Authors:** Koshi Nagai, Baasanjav Uranbileg, Zhen Chen, Amane Fujioka, Takahiro Yamazaki, Yotaro Matsumoto, Hiroki Tsukamoto, Hitoshi Ikeda, Yutaka Yatomi, Hitoshi Chiba, Shu‐Ping Hui, Toru Nakazawa, Ritsumi Saito, Seizo Koshiba, Junken Aoki, Daisuke Saigusa, Yoshihisa Tomioka

**Affiliations:** ^1^ Laboratory of Oncology, Pharmacy Practice and Sciences, Graduate School of Pharmaceutical Sciences Tohoku University Sendai Japan; ^2^ Department of Clinical Laboratory Medicine University of Tokyo Japan; ^3^ Faculty of Health Science Hokkaido University Japan; ^4^ Department of Ophthalmology Tohoku University Graduate School of Medicine Sendai Miyagi Japan; ^5^ Tohoku University Advanced Research Center for Innovations in Next-Generation Medicine; ^6^ Department of Integrative Genomics Tohoku University Tohoku Medical Megabank Organization Sendai Japan; ^7^ Medical Biochemistry Tohoku University Graduate School of Medicine Sendai Japan; ^8^ Laboratory of Molecular and Cellular Biochemistry, Graduate School of Pharmaceutical Sciences Tohoku University Sendai Japan

## Abstract

**Rationale:**

Hepatocellular carcinoma (HCC) is a highly malignant disease for which the development of prospective or prognostic biomarkers is urgently required. Although metabolomics is widely used for biomarker discovery, there are some bottlenecks regarding the comprehensiveness of detected features, reproducibility of methods, and identification of metabolites. In addition, information on localization of metabolites in tumor tissue is needed for functional analysis. Here, we developed a wide‐polarity global metabolomics (G‐Met) method, identified HCC biomarkers in human liver samples by high‐definition mass spectrometry (HDMS), and demonstrated localization in cryosections using desorption electrospray ionization MS imaging (DESI‐MSI) analysis.

**Methods:**

Metabolic profiling of tumor (*n* = 38) and nontumor (*n* = 72) regions in human livers of HCC was performed by an ultrahigh‐performance liquid chromatography quadrupole time‐of‐flight MS (UHPLC/QTOFMS) instrument equipped with a mixed‐mode column. The HCC biomarker candidates were extracted by multivariate analyses and identified by matching values of the collision cross section and their fragment ions on the mass spectra obtained by HDMS. Cryosections of HCC livers, which included both tumor and nontumor regions, were analyzed by DESI‐MSI.

**Results:**

From the multivariate analysis, *m*/*z* 904.83 and *m*/*z* 874.79 were significantly high and low, respectively, in tumor samples and were identified as triglyceride (TG) 16:0/18:1(9*Z*)/20:1(11*Z*) and TG 16:0/18:1(9*Z*)/18:2(9*Z*,12*Z*) using the synthetic compounds. The TGs were clearly localized in the tumor or nontumor areas of the cryosection.

**Conclusions:**

Novel biomarkers for HCC were identified by a comprehensive and reproducible G‐Met method with HDMS using a mixed‐mode column. The combination analysis of UHPLC/QTOFMS and DESI‐MSI revealed that the different molecular species of TGs were associated with tumor distribution and were useful for characterizing the progression of tumor cells and discovering prospective biomarkers.

## 1 INTRODUCTION

Hepatocellular carcinoma (HCC), the primary type of liver cancer, is highly malignant and is continuing to increase worldwide. The five‐year survival rate is less than 15%, and HCC is the second leading cause of death in East Asia.^1^ Early diagnosis of HCC is necessary to reduce the number of deaths and maintain quality of life with cancer. α‐Fetoprotein (AFP) and a protein induced by vitamin K absence or antagonist‐2 (PIVKA‐II) are conventionally used as diagnostic markers for HCC. However, the number of new cases of HCC is currently equal to the number of deaths every year, and these diagnostic markers might not be reflected in the early stage of HCC.^2^, ^3^ Therefore, it is necessary to develop prospective or prognostic biomarkers to characterize the progression of HCC in relation to the phenotype of tumor cells.

The phenotypic changes in HCC are regulated by the genome, epigenome, transcriptome, proteome, environmental factors, and microenvironment, such as growth factors and cytokines,^4^, ^5^, ^6^ and are extraordinarily heterogeneous among individuals.^7^ Metabolomics is the field of omics studies that examines a whole set of small molecules in biological samples and is able to detect subtle changes in metabolic pathways and deviations from homeostasis before the manifestation of phenotype. Metabolomics is therefore widely used for biomarker discovery and is a promising approach for the identification of disease‐related small molecules.^8^, ^9^ Therefore, metabolomics, also called metabolic phenotyping, is an approach with the potential to discover biomarker candidates for HCC by analyzing tumor expression.

Metabolomics is classified into targeted metabolomics (T‐Met) and untargeted metabolomics.^10^ Although the quantitative values of known features of a metabolic pathway can be acquired and analyzed with other omics layers by T‐Met,^11^, ^12^ it is possible to miss the metabolite most associated with a disease. On the other hand, untargeted metabolomics can profile the phenotype based on a number of features and unbiased analysis to identify the disease‐related metabolites in the process of biomarker discovery^13^ and can be useful in searching for disease markers and elucidating biological function.^14^, ^15^ Although the value of untargeted metabolomics has been recognized in the clinical field,^16^ some bottlenecks remain to be considered.

Multiple analytical platforms were conventionally prepared for untargeted metabolomics to acquire a wide polarity range of metabolites as for global metabolomics/metabolic profiling (G‐Met) which were time‐consuming for acquisition and data analysis.^17^ Although G‐Met has already been applied for large‐scale analyses such as cohort studies,^18^, ^19^ the median intensities of detected metabolites decreased during the assays along with the injections required for normalization.^17^ Therefore, a comprehensive and reproducible method is needed for G‐Met in one assay. Recently, a mixed‐mode column including both an ion‐exchange phase and a reverse phase was developed, allowing a wide polarity range of metabolites to be separated in a short time in one assay.^20^, ^21^

The metabolites were generally identified by the matching of mass spectra and fragment ions with high accuracy to theoretical values in a database and matching of the retention times of the chromatogram to chemical standards,^22^, ^23^ which defined the quality of identification.^24^, ^25^ However, there were still isomeric and isobaric species, such as lipids. High‐definition mass spectrometry (HDMS) is a technology that relies on the mobility of ions, including small molecules, according to the charge, shape, and size in the gas phase inside the MS instrument.^26^, ^27^ The collision cross section (CCS) of each metabolite can be calculated with the drift time acquired in the mobility cell to provide specific information on each structure. In addition, the background noise levels were reduced using HDMS, and clear mass spectra could be obtained for the identification of metabolites.^28^, ^29^, ^30^

In the study reported here, we developed a wide‐polarity G‐Met method using a mixed‐mode column and identified biomarker candidates for HCC in human liver samples using HDMS. Then, we evaluated the biomarkers by determining their localization in tumor tissue sections using desorption electrospray ionization (DESI) MS imaging (MSI).

## 2 EXPERIMENTAL

### 2.1 Materials

Methanol and 2‐propanol of LC/MS grade were purchased from Kanto Chemical (Tokyo, Japan) and Thermo Fisher Scientific (Waltham, MA, USA), respectively. Ammonium formate (1 mol/L) and formic acid of LC/MS grade were purchased from Fujifilm Wako Chemical Industries (Osaka, Japan). For synthesis of triglyceride (TG) standard, 1‐palmitoyl‐sn‐glycerol (MG 16:0/0:0/0:0), 11(*Z*)‐eicosenoic acid (20:1(9*Z*)), and oleic acid (18:1(9*Z*)) were purchased from Sigma‐Aldrich Japan (Tokyo, Japan). Other chemicals and reagents of analytical grade were purchased from Wako Pure Chemical Industries Ltd (Osaka, Japan) unless otherwise specified.

### 2.2 Samples

Institute of Cancer Research mouse liver tissue was purchased from KAC Co. (Kyoto, Japan). Human liver was obtained from HCC patients at the Hepatobiliary Pancreatic Surgery Division, Department of Surgery, at the University of Tokyo Hospital between January 2013 and October 2014. Patient information was described in a previous report.^4^ The research ethics committee of the Faculty of Medicine, University of Tokyo approved the present study, which was conducted in accordance with the ethical guidelines of the 1975 Declaration of Helsinki. All patients provided written informed consent for the use of clinical samples.

### 2.3 Preparation of mouse liver tissue samples

Liver samples of approximately 50 mg each from male or female mice at 4, 6, or 8 weeks old were prepared (*n* = 8, each). Methanol containing 0.1% formic acid (10 μL/mg tissue) was added to the samples. After homogenization with beads at 5500 rpm for 20 s (2.8 mm zirconium oxide beads) and sonication in an ultrasonic bath for 10 min, the samples were centrifuged at 16 400 × *g* for 20 min at 4°C. Then, the supernatant was transferred to a 1.5‐mL sample tube and diluted (×2) with water containing 0.1% formic acid. Eight samples were mixed and transferred to eight positions of a 96‐well plate (700 μL round well; Waters Corp., Milford, MA, USA). The preparation of study quality control (SQC) and dilution quality control (dQC) and the run order of samples for G‐Met analysis were performed as described in a previous report.^17^ Then, 4‐μL samples were injected into an ultrahigh‐performance liquid chromatography quadrupole time‐of‐flight MS (UHPLC/QTOF/MS) system.

### 2.4 UHPLC/QTOFMS analysis

The UHPLC/QTOFMS system consisted of an ACQUITY UPLC instrument (Waters Corporation, Milford, MA, USA) and a Synapt G2‐Si QTOF mass spectrometer (Waters Corporation, Manchester, UK) and was operated in both positive and negative ion mode for electrospray ionization (ESI). The capillary voltage, cone voltage, source temperature, desolvation temperature, and desolvation gas flow rate in positive ion mode were 2.0 kV, 20 V, 150°C, 500°C, and 1000 L/h, respectively. The desolvation temperature and desolvation gas flow rate in negative ion mode were 600°C and 1200 L/h, respectively.

LC separation was performed using a Scherzo SS‐C18 mixed‐mode column (2.0 mm i.d. × 50 mm, 3 μm particle size; Imtakt, Kyoto, Japan). The mobile phases consisted of water containing 0.1% formic acid (A), methanol/2‐propanol/ammonium formate (1 mol/L), 50/50/3 (v/v/v%) (B), and methanol (C). Features were separated by gradient conditions; the initial condition was 0% B with 0.4 mL/min, followed by a linear gradient to 100% B from 1 to 4 min; 100% B was maintained for 2.5 min; and the mobile phase was returned to the initial conditions and maintained for 2.5 min until the end of the run. The total run time was 10 min, and the column oven temperature was 45°C. The flow line of the mobile phase (C) was connected to the line from the column to the ESI source as the postcolumn infusion.

### 2.5 Data processing

The data procession of G‐Met was followed as in a previous report.^17^ All data obtained using UHPLC/QTOFMS were imported to Progenesis QI 3.0.3.0 (Nonlinear Dynamic, Newcastle, UK) for peak picking, alignment, and normalization to produce peak intensities for retention time (*t*_R_) and *m*/*z* data pairs. The relative intensities of features were analyzed by principal component analysis (PCA) using SIMCA13.0.0 (Umetrics, Umeå, Sweden), and normalized with Quantbolome software.

### 2.6 Human liver analysis by UHPLC/QTOFMS

Tumor (*n* = 39) and nontumor (*n* = 79) human liver samples were prepared as described in section 2.3. The supernatant of the centrifuged sample was transferred to a 96‐well sample collection plate and diluted (×2) on the plate with water containing 0.1% formic acid. The preparation of SQC and dQC and the run order of samples for G‐Met analysis were performed as described in a previous report.^17^ Then, 4‐μL samples were injected into the UHPLC/QTOFMS system. The data processing was performed as described above. Then, the normalized intensities of features were used for further multivariate analysis, such as PCA and orthogonal partial least squares discriminant analysis (OPLS‐DA).

### 2.7 Identification of metabolites by HDMS

The features extracted by OPLS‐DA were first identified with the Chemspider database, human metabolome database, and Lipidmaps database. Then, the features were identified with the Synapt G2‐Si (traveling‐wave ion mobility mass spectrometer) system in HDMS mode. The source parameters for ESI were the same as described in section 2.4. The start and end of the wave velocity were 1000 and 300 m/s, respectively. The wave height, bias, and step wave voltages were 40, 45 and 10 V, respectively. The CCS value was calculated automatically using ProgenesisQI, and the fragment ion mass spectra using HDMS were obtained at the same time. Each identified metabolite was purchased or synthesized in the laboratory, and the chemical standard solution was analyzed by HDMS.

### 2.8 Synthesis of TG 16:0/18:1(9*Z*)/20:1(11*Z*)

The synthetic schemes of TG 16:0/18:1(9*Z*)/20:1(11*Z*) were according to our previously published method.^31^ Briefly, MG 16:0/0:0/0:0 was first esterified with fatty acid (FA) 20:1(9*Z*) in anhydrous pyridine for 2 h at 0°C, followed by purification with a silica gel column to yield diglyceride 16:0/0:0/20:1(11*Z*) (yield 27.6%). Then, the second esterification was performed by adding FA 18:1(9*Z*) in anhydrous pyridine for 4 h at room temperature. After purification with a silica gel column, the TG 16:0/18:1(9*Z*)/20:1(11*Z*) was obtained as colorless oil (yield 73.6%). ^1^H NMR (CDCl_3_, *δ*): 0.86 (*t*, *J* = 6.5 Hz, 9H, —CH_3_), 1.18–1.39 (*m*, 68H, —CH_2_—), 2.00 (*m*, 8H, —C=C—CH_2_—), 2.24–2.36 (*m*, 6H, —OCO—CH_2_—), 4.14 (*dd*, *J* = 11.8 and 5.9 Hz, 2H, 1‐H and 3‐H), 4.29 (*dd*, *J* = 11.6 and 4.0 Hz, 2H, 1‐H and 3‐H), 5.26 (*tt*, *J* = 5.9 and 4.4 Hz, 1H, 2‐H), and 5.29–5.39 ppm (*m*, 4H, —CH=CH—), data which were in accordance with a previous study.^31^

### 2.9 Distribution analysis using DESI‐MSI

Cryosections (20 μm in thickness) of human liver, including both tumor and nontumor regions, were cut by a cryostat, placed on a normal glass slide, inserted into a 50‐mL tube with drying silica gel, and stored at −30°C. DESI‐MSI analysis was performed with the Synapt G2‐Si QTOF mass spectrometer. Then, the sample on the glass slide was set on a two‐dimensional moving stage, and the sections were analyzed by DESI‐MSI in positive ion mode over the range *m*/*z* 50 to 1200. The DESI spray solvent, solvent flow rate, and spray gas (nitrogen) pressure were methanol containing 2% water, 2 μL/min, and 0.4 MPa, respectively. The spatial resolution, stage rate, scan time, and total time of analysis were 100 μm, 100 μm/s, 1 s, and 240 min, respectively. The capillary voltage, cone voltage, and source temperature for ionization were 5.0 kV, 50 V, and 150°C, respectively. The 1000 highest‐intensity peaks of the ions in the *m*/*z* range were obtained from the whole regions of cryosection in High Definition Imaging (HDI) software (Waters Corporation) to process the mass spectral data and to construct two‐dimensional ion images.

## 3 RESULTS AND DISCUSSION

### 3.1 UHPLC/QTOFMS analysis

**Figure 1:**
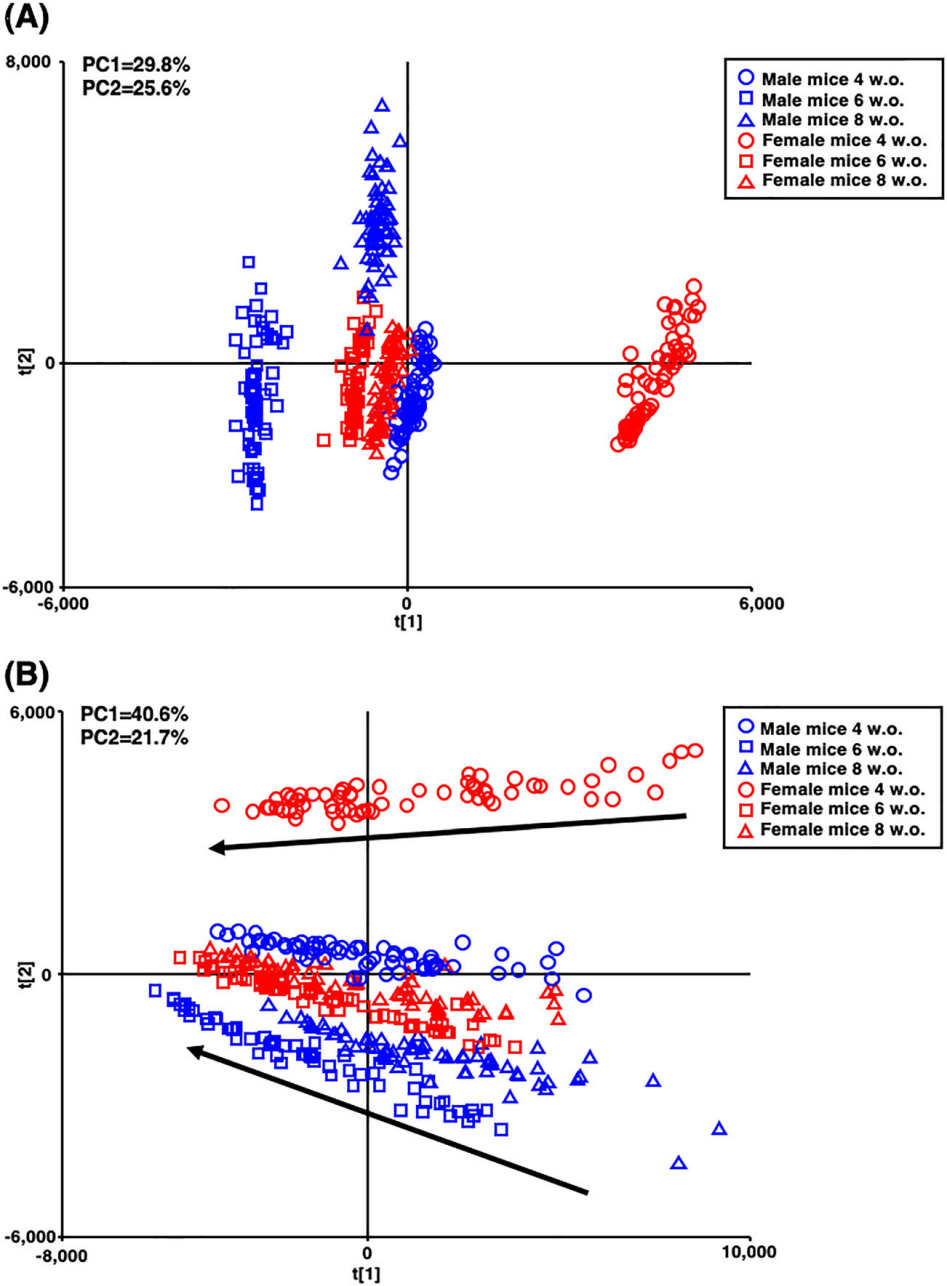
PCA score plot based on the intensity of features detected in mouse liver A, after and B, before normalization. Four‐, six‐, and eight‐week‐old male mice are represented by blue circles, squares, and triangles, and female mice are represented by red circles, squares, and triangles, respectively. The time‐dependent drifts of the sample analyses are shown by black arrows [Color figure can be viewed at http://wileyonlinelibrary.com]

We first evaluated the utility of G‐Met by UHPLC/QTOFMS with a mixed‐mode column for the analysis of multiple biological liver samples. The six groups of mice were clearly separated on the PCA score plot based on the detected chemical features from mouse livers after normalization by software (Figure 1A). The median intensities of ions decreased with the analysis of multiple injections,^32^ as we could see the time‐dependent drift line on the score plot of PCA before normalization (Figure 1B, black arrows). The results indicated that the present method can be utilized for the G‐Met analysis of liver samples with the normalization procedure.

### 3.2 Human liver analysis by UHPLC/QTOFMS

**Figure 2:**
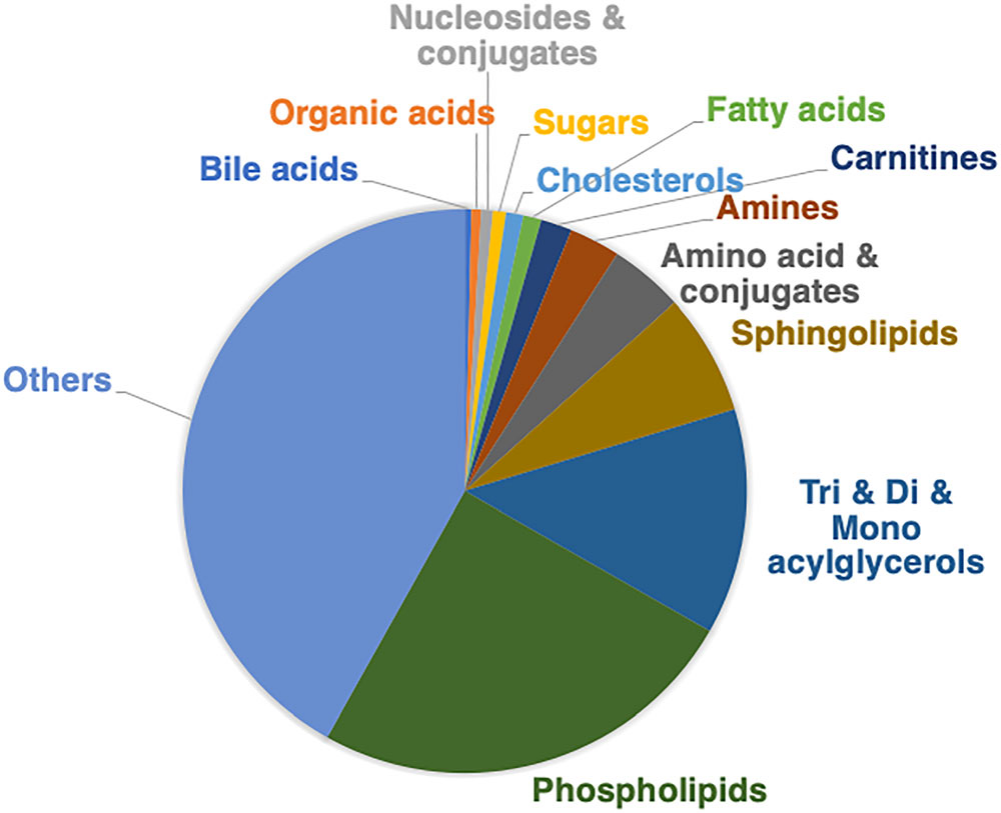
Classification of 2122 metabolites in human liver detected by UHPLC/QTOFMS analysis with the mixed‐mode column [Color figure can be viewed at http://wileyonlinelibrary.com]

We then applied the G‐Met method to human tumor and nontumor liver samples. A total of 2447 or 1601 features were originally detected in positive or negative ion mode, and 1394 or 728 metabolites were identified with the databases within 5 ppm mass accuracy and classified into 13 groups, which included candidates based on the biological function of metabolites or lipid species, as shown in Figure 2. Although G‐Met has been performed with a hydrophilic interaction liquid chromatography column and/or a reversed‐phase liquid chromatography C18 column, each method has a limited capacity to retain hydrophobic and/or hydrophilic metabolites, respectively, and decreased reproducibility because of ion suppression for those metabolites.^33^ Therefore, combination analyses by both columns have been conventionally used for G‐Met. The mixed‐mode column combines ionic phases with the C18 phase and improves the retention of both hydrophilic and hydrophobic metabolites.^20^ The present G‐Met method that we developed can expand the polarity range of detected metabolites in one assay and has enough coverage of biological molecules for biomarker discovery.

**Figure 3:**
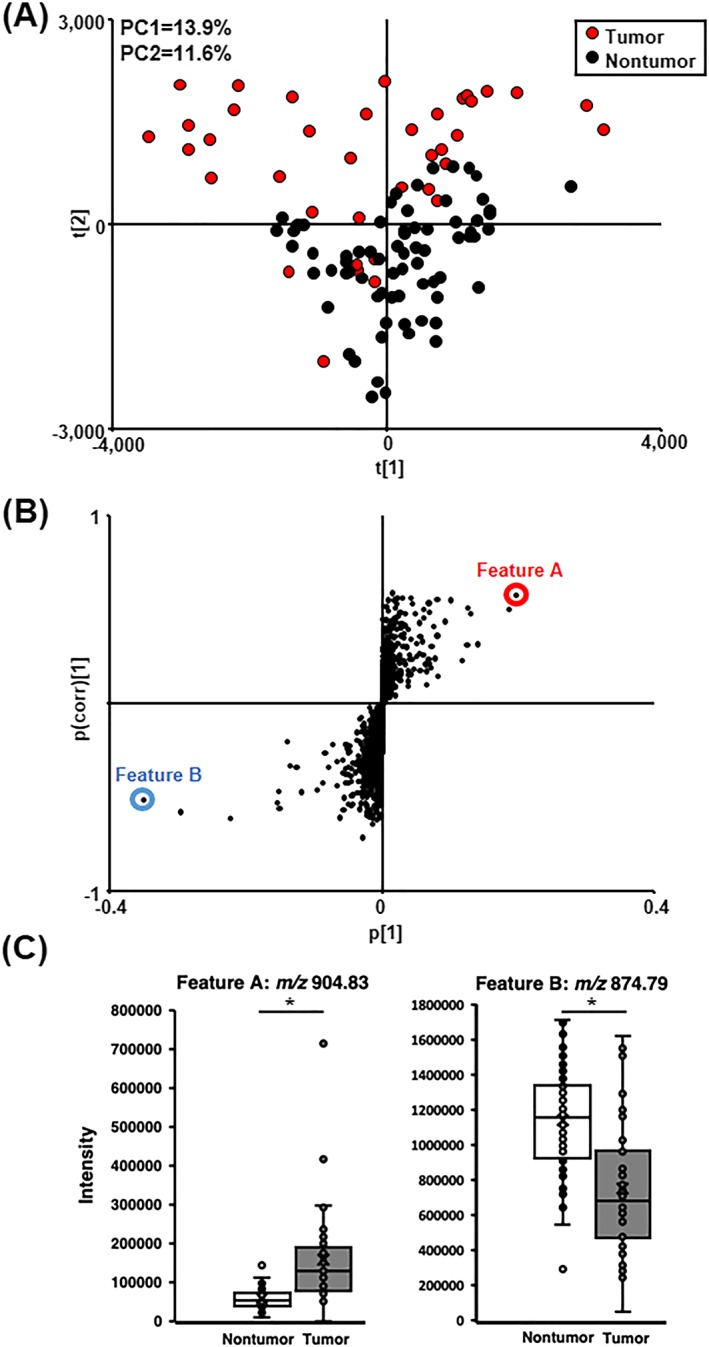
A, PCA score plot based on the intensity of features in human liver. The nontumor and tumor samples are represented by black and red dots. B, *S*‐plot of OPLS‐DA between nontumor and tumor tissue of human livers. All features are represented by black dots, and the features with the highest contributions, namely with higher (feature A) and lower (feature B) values in tumor tissue, are marked by red and blue circles, respectively. C, Box plots of the intensity of feature A: *m*/*z* 904.83; and feature B: *m*/*z* 874.79 in nontumor and tumor tissue from human liver. Boxes represent the interquartile range (IQR) between the first (Q1) and third quartiles (Q3); the line inside represents the median. Whiskers denote the lowest and highest values within 1.5 × IQR from Q1 and Q3, respectively. Statistical significance (*p < 0.01) determined by Mann–Whitney U test [Color figure can be viewed at http://wileyonlinelibrary.com]

We next compared the intensity of 2447 features originally detected in positive ion mode from the tumor and nontumor regions by using multivariate analyses. The tumor regions were slightly shifted from the nontumor regions and overlapped on the PCA score plot (Figure 3A). We then extracted the features that contributed to the separation on the *S*‐plot of OPLS‐DA, as shown in Figure 3B. Feature A (*m*/*z* 904.83, marked in red) and feature B (*m*/*z* 874.79, marked in blue) were significantly different in the tumor regions and were selected as candidates for HCC biomarkers (Figure 3C). On the other hand, citric acid was significantly higher in the tumor regions while any other specific metabolites could not be extracted by the multivariate analyses in negative ion mode. The citrate synthesis was upregulated in HBV replication and the enzymes of the tricarboxylic acid cycle were promoted,^34^ and the phenomenon is well known as the mitochondrial metabolism in tumor. We thus decided to proceed with the identification of novel biomarkers in the positive ion mode analyses.

### 3.3 Identification of metabolites by HDMS

Features A and B were identified as species of TG in the database; however, a number of candidates were still included on the list. We therefore injected the sample into a UHPLC/QTOFMS system with HDMS mode. The signal intensities of ions are generally decreased in HDMS mode because of the loss of ions during ion mobility separation.^35^ Therefore, methanol was added at a flow rate of 0.2 mL/min in the line from the outlet of the column to the ESI source to increase the capacity of ionization.

**Figure 4:**
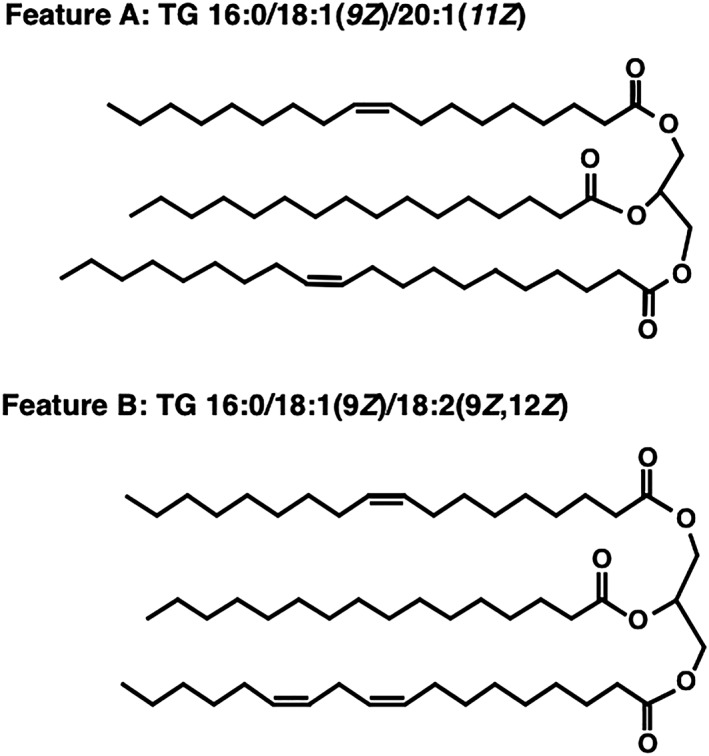
Chemical structures of feature A: TG 16:0/18:1(9*Z*)/20:1(11*Z*); and feature B: TG 16:0/18:1(9*Z*)/18:2(9*Z*,12*Z*)

The CCS values of feature A and B obtained by HDMS were 338.7 and 326.1 and matched the values of TG (54:2) [M + NH_4_]^+^ and TG (52:2) [M + NH_4_]^+^, respectively, in a previous report.^36^ The acceptable difference in the CCS value (ΔCCS) has previously been defined at less than 2%,^37^ and the ΔCCS of features A and B were 1.4% and 0.3%, respectively, which were satisfactory values. In addition, the fragment ions obtained by MS^E^ were clearer on the mass spectrum obtained by HDMS, and the fragmentation scores of features A and B were 99.4 and 96, respectively. Features A and B were finally identified as TG 16:0/18:1(9*Z*)/20:1(11*Z*) and TG 16:0/18:1(9*Z*)/18:2(9*Z*,12*Z*), respectively, and their structures are shown in Figure 4.

We then synthesized the chemical standard TG 16:0/18:1(9*Z*)/20:1(11*Z*) described in section 2.8 and purchased TG 16:0/18:1(9*Z*)/18:2(9*Z*,12*Z*) to confirm their structures. The CCS values and fragment ions of the chemical standards are completely consistent with those for liver samples.

Several candidates for HCC biomarkers currently exist; however, most have not been identified through the requisite phases of biomarker development, and there is a lack of clear data regarding the selection of optimum agents for individual patients.^38^ Therefore, novel biomarkers are needed to enable early diagnosis and personalized therapy for HCC, and it is necessary to study the function of molecules that can predict disease progression or prognosis to improve clinical outcomes.^39^

### 3.4 Distribution analysis using DESI‐MSI

Although biomarker candidates were identified with G‐Met analysis using UHPLC/QTOFMS, their localized information was lost in the tissue sample. MSI can directly analyze small molecules in a tissue section and observe their localization.^40^ MSI has the potential to overcome the disadvantages of G‐Met and has already been widely applied for cancerous tissues using the traditional matrix‐assisted laser desorption ionization technique.^41^, ^42^ Recently, DESI‐MSI was also applied as a matrix‐free approach to the imaging of small molecules in tissue samples,^39^, ^43^ and it might be possible to obtain similar corresponding profiles with UHPLC/QTOFMS analysis because of ESI‐based ionization.

**Figure 5:**
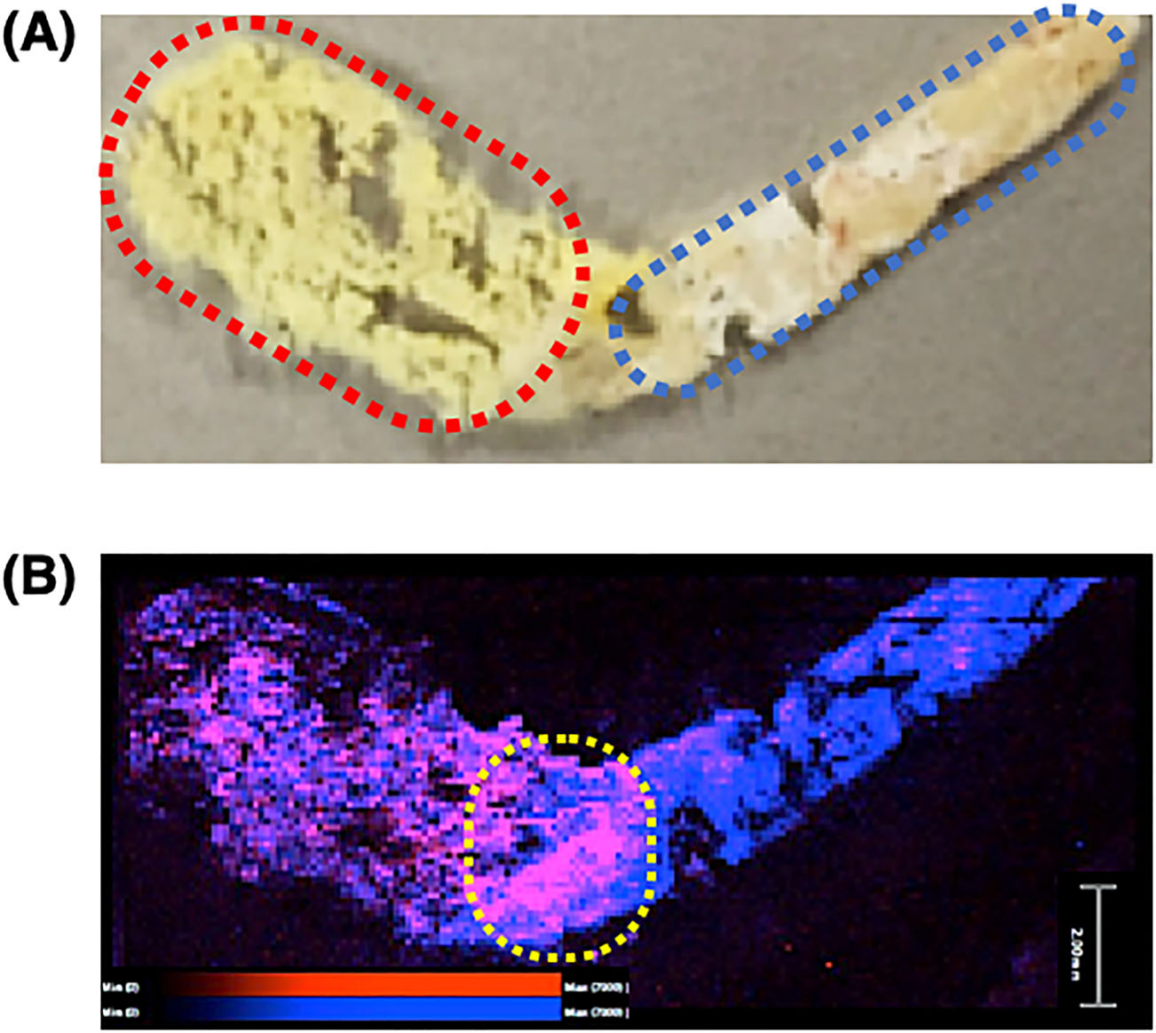
A, Optical image of cryosection obtained from an HCC human liver tissue sample. The tumor and nontumor regions are marked with red and blue dotted lines, respectively. B, Merged image of TG 16:0/18:1(9*Z*)/20:1(11*Z*): *m*/*z* 904.83; and TG 16:0/18:1(9*Z*)/18:2(9*Z*,12*Z*): *m*/*z* 874.79 detected by DESI‐MSI analyses on the cryosection obtained from a human liver tissue sample of HCC. TG 16:0/18:1(9*Z*)/20:1(11*Z*) and TG 16:0/18:1(9*Z*)/18:2(9*Z*,12*Z*) are represented by red and blue, respectively. The boundary region of the tumor and nontumor is marked by a yellow dotted circle [Color figure can be viewed at http://wileyonlinelibrary.com]

We then examined the DESI‐MSI analysis using an HCC liver tissue sample including both tumor and nontumor regions, which are marked by red and blue dashed areas on the optical image, respectively (Figure 5A), to evaluate the localization of the TGs. The ions identified with TG 16:0/18:1(9*Z*)/20:1(11*Z*) (*m*/*z* 904.83, red colored) and TG 16:0/18:1(9*Z*)/18:2(9*Z*,12*Z*) (*m*/*z* 874.79, blue colored) were localized in the tumor region and the nontumor region, respectively, by DESI‐MSI analysis (Figure 5B). The species of FA of TG 16:0/18:1(9*Z*)/20:1(11*Z*) consists of saturated fatty acid (SFA) and monounsaturated fatty acids (MUFAs), while polyunsaturated fatty acid (PUFA) is included in the structure of TG 16:0/18:1(9*Z*)/18:2(9*Z*,12*Z*). Several studies have already reported differences in the species of TG in tumor tissues.^44^, ^45^, ^46^ The major species of SFA and MUFA, palmitic acid (C16:0) and oleic acid (C18:1), were higher in the tumor region, and PUFA, including linoleic acid (C18:2), was lower in the tumor region than in the nontumor region,^47^, ^48^ which was consistent with our results.

These TGs showed an interesting overlap at the boundary region, which is marked by a yellow dotted circle in Figure 5B. Muir et al reported that the expression of fatty acid synthase (*Fasn*), fatty acid elongase (*Elovl1*, *Elovl6*), fatty acid desaturase (*Fads*), and stearoyl‐CoA desaturase (*SCD*) was increased by nonalcoholic steatohepatitis, which is a preneoplastic condition in tumor status.^49^ Additionally, the single‐nucleotide polymorphisms related to the gene expression of SCD were correlated with HCC malignancy in the cohort analysis and might indicate that several backgrounds in FA metabolism can contribute to the expression of HCC.^50^ Although the FA species of TG are related to HCC progression and malignancy according to previous and present studies, the direct function of differences in carbon chain length or the number of double bounds is still unknown, and further analysis is necessary to clarify the physiological function of several metabolic pathways related to TG metabolism in tumors.

Recently, the identification of tumor and nontumor regions was achieved using direct MS technology in the tissue of cancer patients during surgical operation.^51^ The profiling of FA species in TG has the potential to distinguish tumor, nontumor, and boundary regions by means of this technology. However, the quality of biomarker candidates generally must be evaluated by plasma analysis to be used in clinical diagnosis. AFP is conventionally used for the diagnosis of HCC and shows 60%–80% sensitivity as a biomarker. However, the sensitivity decreases to approximately 40% for the detection of small tumors.^52^ Therefore, the combination of several molecules is necessary to improve the sensitivity and specificity for the early diagnosis of HCC.^38^ In this study, we demonstrated the difference in TG species in HCC tissue. However, it is still necessary to evaluate the correlation with the plasma concentration of TGs and reproducibility as a candidate biomarker with increased sample numbers.

## 4 CONCLUSIONS

The present G‐Met method with a mixed‐mode column was comprehensive and reproducible and could detect a wide polarity range of metabolites in biological samples. Novel biomarkers for HCC were identified with the G‐Met method, and the difference in FA species of TG in tumor regions was demonstrated by HDMS in combination with UHPLC/QTOFMS and DESI‐MSI. We thus conclude that G‐Met by a combination of UHPLC/QTOFMS with HDMS and distribution analysis by DESI‐MSI is useful for characterizing the progression of tumor cells and discovering prospective biomarkers.
